# Supplementary material for: Phosphatidic Acid Accumulates at Areas of Curvature in Tubulated Lipid Bilayers and Liposomes
Source: Biomolecules. 2022 Nov 17;12(11):1707. doi: 10.3390/biom12111707 (PMC9687397; doi:10.3390/biom12111707)
Supplement: Supplementary file 1 [file biomolecules-12-01707-s001.zip › biomolecules-1971922-supplementary.pdf]

Supplementary Data

Phosphatidic Acid Accumulates at Areas of Curvature in Tubulated Lipid Bilayers and Liposomes

Broderick L. Bills, Michelle K. Knowles*
Department of Chemistry and Biochemistry, University of Denver, Denver, CO 80210
Molecular and Cellular Biophysics Program, University of Denver, Denver, CO 80210

Supplementary Methods:

Melittin quenching of NBD liposomes: Liposomes containing 98% POPC, 1% DOPE-PEG and 1% of either 18:1 12:0 NBD PA or 18:1 12:0 NBD PC in 2.5 mL buffer (140 mM KCl, 20 mM HEPES and 15mM NaCl at pH 7.4) were extruded through 100 nm filters and put in a 1 cm quartz cuvette with a stir bar. 250 μL of 0.1 mM dithionite was added after 30 s and 1.76 μM of melittin (Sigma Aldrich) was added at 120 s to form pores. Readings were taken on a Cary Eclipse once per second, with 0.1 second exposure, an excitation wavelength of 463 nm and emission of 533 nm.

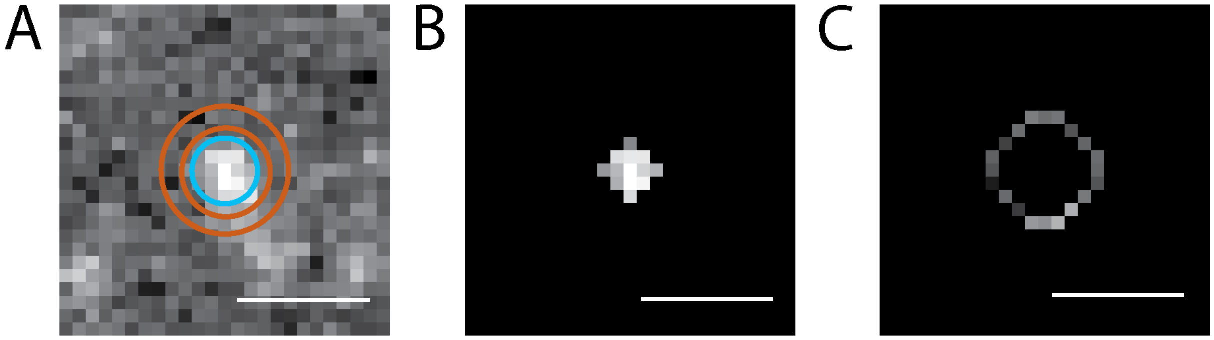

Supplemental Figure S1: Description of ΔF/S Measurements. A) Depiction of the circle (blue) and annulus (orange) regions overlaid on the image of a tubule in the DiD (red) channel. There is a 1-pixel thick ring that separates the circle and annulus regions.. B) Example of the same tubule with the circle intensity shown. The average of this region constitutes the circle intensity. C) Example of the same tubule with the annulus region intensity shown. The average of this ring constitutes the annulus intensity. The intensities of the circle and annulus are obtained and used to calculate ΔF/S, where ΔF = circle – annulus and S = annulus – background, where background is the average intensity of a POPC membrane with no fluorophores present. Scale bars = 1 μm.

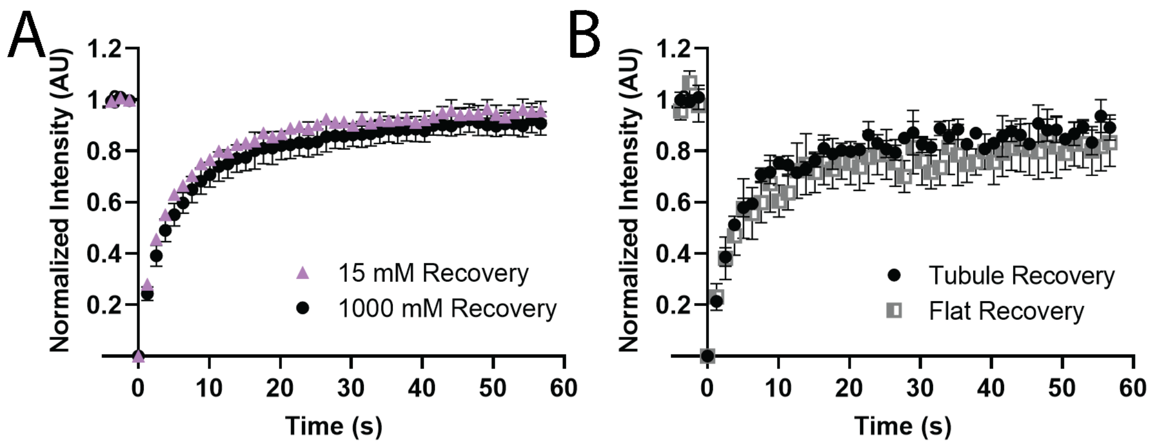

Supplemental Figure S2: STuBs Do Not Affect Fluidity of MB-DHPE. A) FRAP recovery of SLBs deposited in 15 mM NaCl buffer (purple triangles) or 1000 mM NaCl buffer (black circles). B) FRAP recovery of tubules (black circles) or flat regions (grey squares) specifically. For both graphs, intensity was normalized for photobleaching and such that 1 is the average of three pre-FRAP frames and is the frame immediately after FRAP. Error bars are SEM (n = 3).

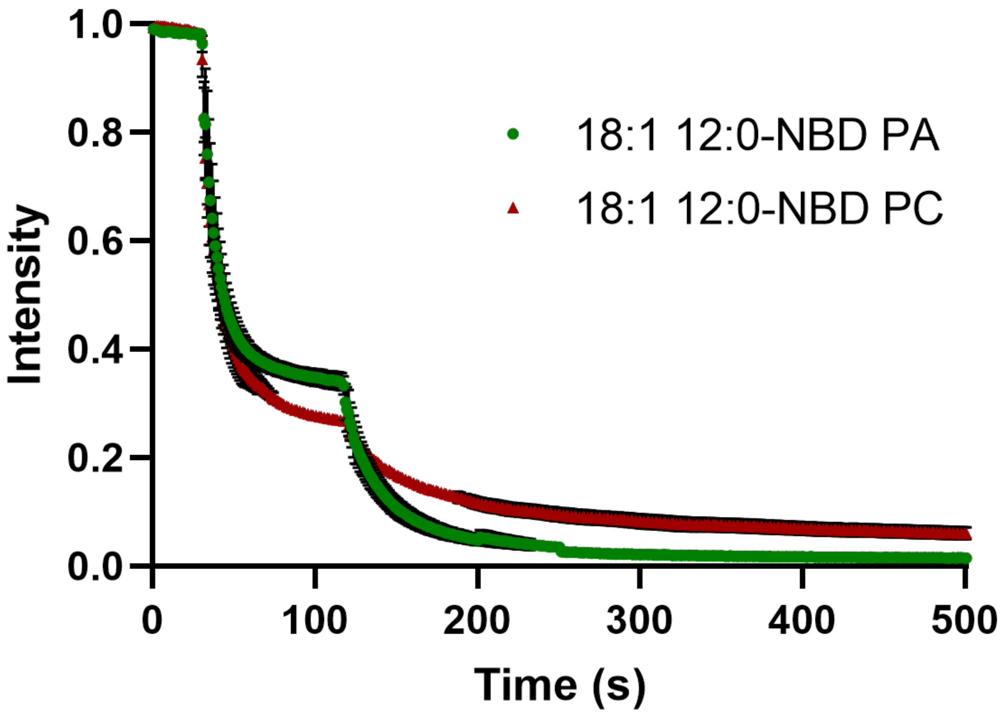

Supplemental Figure S3: Dithionite and Melittin Fluorimetry Traces of 18:1 12:0-NBD PA and PC. Normalized intensity trace of NBD-PC (red triangles) and NBD-PA (green squares), where dithionite was added at 30 seconds and melittin was added at 120 seconds to a final concentration of 1.76 μM. Error bars are SEM for three independent replicates and often are smaller than the data points.
